# High rates of chronic HBV genotype E infection in a group of migrants in Italy from West Africa: Virological characteristics associated with poor immune clearance

**DOI:** 10.1371/journal.pone.0195045

**Published:** 2018-03-29

**Authors:** Vincenzo Malagnino, Romina Salpini, Gaetano Maffongelli, Arianna Battisti, Lavinia Fabeni, Lorenzo Piermatteo, Luna Colagrossi, Vanessa Fini, Alessandra Ricciardi, Cesare Sarrecchia, Carlo Federico Perno, Massimo Andreoni, Valentina Svicher, Loredana Sarmati

**Affiliations:** 1 Clinical Infectious Diseases, Department of System Medicine, Tor Vergata University, Rome, Italy; 2 Department of Experimental Medicine and Surgery, Tor Vergata University, Rome, Italy; Universita degli Studi di Pisa, ITALY

## Abstract

Hepatitis B virus (HBV) genotype E almost exclusively occurs in African people, and its presence is more commonly associated with the development of chronic HBV (CHB) infection. Moreover, an epidemiological link has been found between the distribution of HBV genotype E infection and African countries with high incidences of hepatocellular carcinoma. As part of a programme for the health assessment of migrants, we evaluated 358 young African subjects for HBV infection; 58.1% (208/358) were positive for an HBV marker, and 54 (25.5%) had CHB. Eighty-one percent of the CHB subjects were infected with HBV genotype E, with a median serum HBV-DNA of 3.2 (IQR: 2.7–3.6) logIU/ml. All patients had high serum HBsAg titres (10,899 [range 5,359–20,272] IU/ml), and no correlation was found between HBsAg titres and HBV-DNA plasma levels. RT sequence analysis showed the presence of a number of immune escape mutations: strains from all of the patients had a serine at HBsAg position 140; 3 also had T116N, Y100C, and P142L+S143L substitutions; and 1 had a G112R substitution. Six (18%) patients had stop-codons at position 216. In 5 of the 9 (26.5%) CHB patients, ultrasound liver biopsy, quantification of total intrahepatic HBV-DNA and cccDNA, and RT/HBsAg sequencing were performed. The median (IQR) total intrahepatic HBV-DNA was 766 (753–1139) copies/1000 cells, and the median (IQR) cccDNA was 17 (10–27) copies/1000 cells. Correlations were observed for both total intrahepatic HBV-DNA and cccDNA with serum HBV-DNA, while no correlation was found for the HBsAg titres. A difference of 2.5/1,000 nucleotides was found in the HBsAg sequences obtained from plasma and from liver tissue, with 3 cases of possible viral anatomical compartmentalization. In conclusion, a high rate of CHB infection due to the E genotype was demonstrated in a group of immigrants from Western Africa. An analysis of the viral strains obtained showed the virological characteristics of immune escape, which may be the cause of viral replication persistence. Moreover, a fair percentage of stop codon mutations were found. The lack of correlation between HBsAg titres and plasma or intrahepatic HBV-DNA found in these subjects suggests a pathway of virus production that is not linked to HBsAg secretion. Studies with a larger number of patients with CHB due to the E genotype are advisable to corroborate these observations.

## Introduction

At least 2 billion people have been infected with hepatitis B virus (HBV) worldwide, and approximately 360 million patients have a chronic HBV infection (CHB), which appears to cause 50% to 60% of the hepatocellular carcinoma (HCC) cases [1] worldwide.

Africa is considered a region with high endemicity as it has an estimated Hepatitis B S antigen (HBsAg) seroprevalence of 6–20%, which is higher in rural than in urban areas. HBsAg seroprevalence can first be detected in African children 1–3 years old, and it increases with age, growing to 12–16% in 6 to 15-year-old African adolescents [2].

HBV genotypes A, D and E are the most frequent genotypes found in Africa. Genotype A is found predominantly in Southern, Eastern and Central Africa, while genotype D is most common in Northern Africa. Conversely, HBV genotype E, first described in 1992, predominates in Western Africa and has not been detected outside of Africa [2]. It has been hypothesized that HBV genotype E was introduced into the African population only within the past 200 years, probably through cross-species transmission [3].

A paucity of information is available on the clinical and virological characteristics of HBV genotype E-infected patients. Epidemiological studies have suggested the carcinogenic potential of genotype E, and in fact, African regions in which genotype E is endemic are characterized by a higher incidence of HCC. Recently, an HCC incidence of 55.5/100000 person-years [4] has been reported by analysing 405 chronic HBV carriers in Gambia, 95% of which had a genotype E infection. This incidence is approximately 2-fold higher than that described for HBV genotype A or D patients. Another report from Gambia, where the E genotype is most prevalent, revealed that chronic hepatitis B (CHB) is associated with HCC development at a younger age [5]. In addition, the G1896 mutation in the pre-core region of the HBV genome, which is known to favour HCC onset, frequently occurs in HBV genotype E [6].

A paucity of information is also available on the efficacy of anti-HBV drugs in HBV genotype E-infected patients. In this regard, a recent study has shown poor response to interferon-based treatment in patients with chronic HBV genotype E infection [7].

Cases of HBV genotype E infections have been reported in Europe and in the Americas, usually in migrants from African countries [8–11]. Migration and international travel are considered the main causes that lead to an altered geographical distribution of HBV genotypes around the world, including in low seroprevalence regions such as Europe and North America.

In this study, as part of a programme for the health assessment of migrants admitted to migrant shelters in Rome (Italy), we evaluated 358 young subjects from Africa. Twenty five percent of these individuals were found to have a sustained CHB infection, the large majority of which were caused by HBV genotype E. We provide a description of the clinical and serological features of CHB patients as well as a detailed characterization of the HBV genotype E strains isolated from the peripheral and liver compartments of a subgroup of these subjects.

## Patients and methods

This study included 358 subjects from 10 migrant shelters in Rome (Italy) who received a medical evaluation at the Infectious Diseases Unit at Tor Vergata University Hospital from November 2013 to May 2015. A complete blood count as well as biochemical blood tests and a medical examination were proposed to all subjects. Serological screening for the most common infections was also offered to all of those who agreed to undergo the blood tests. Anti-HIV 1/2, anti-Hepatitis B c antigen (HBc), and anti-Hepatitis C virus (HCV) antibody detection, HBsAg, a Venereal Disease Research Laboratory (VDRL) assay and a QuantiFERON Gold test were proposed as screening tests. All of the subjects who were positive for any of the screening tests were subsequently evaluated for disease staging and possible treatment. All patient personal information was treated in a confidential manner, and all clinical data were collected anonymously to respect patient privacy. All patients who agreed to participate to the study gave written informed consent. All enrolled patients had more than 18 years so in no case a consent to study participation was obtained by parents or guardians. The study protocol was approved by the Ethics Committee.

### Evaluation of HBV-positive patients

All subjects who were HBsAg-positive were then tested for Hepatitis e antigen (HBeAg) and anti-HBeAg, anti-HBcAg, and anti-Hepatitis D virus (HDV) antibodies; HBsAg and HBV-DNA were then quantified, and the HBV genotype was assessed. Serum HBV markers were analysed by commercial immunoenzymatic assays (HBsAg, HBeAg, anti-HBe, anti-HBs, and anti-HBc [total and IgM] from Roche Cobas Diagnostics, North Chicago, IL). Plasma HBV-DNA was quantified by real-time polymerase chain reaction (Roche Cobas^®^ Ampliprep/Cobas^®^ TaqMan). Most patients included in the study had a single point observation for HBV infection since they were in temporary migrant shelters and they moved to other locations after the first visit.

A liver biopsy was proposed to all patients who had a CHB E genotype infection to histologically evaluate liver damage and to define intrahepatic virological parameters. Nine patients gave written informed consent to undergo liver biopsy. For these 9 patients we had the opportunity to have a second follow-up and to further study HBV intrahepatic parameters and liver histology.

#### Population-based sequencing of the HBV Reverse Transcriptase and HBsAg

Population-based sequencing of the HBV Reverse Transcriptase (RT) was performed on plasma and liver samples following previously described protocols [12, 13], the details of which are reported in S1 File. Due to the overlap of the RT and HBsAg genes, the RT sequences were also used to analyse the full-length HBsAg gene. The RT/HBsAg sequences obtained from plasma samples were used to define the HBV genotype and to determine the presence of drug-resistance in the RT as well as immune escape mutations in the HBsAg.

#### Phylogenetic analysis

For each patient, the HBV genotype and sub-genotype were determined. The RT sequences were aligned and compared with reference sequences for all of the HBV genotypes and sub-genotypes [14]. The sequences were then manually edited using the Bioedit programme, and gaps were removed from the final alignment. Genotype assignments were achieved by constructing phylogenetic trees, first using the Neighbor-Joining (NJ) method [15]. Distances were calculated using MEGA 6 based on the Kimura-2 parameter (K2P) model [16]. The robustness of the genotyping assignments was further tested using the Maximum Likelihood (ML) method. The ML tree was inferred using the General Time-Reversible nucleotide substitution model (GTR) with gamma-distribution among site rate heterogeneity, a proportion of invariable sites (GTR+I+Γ5) [17], and 1,000 bootstrap replicates (using the PhyML programme, available at http://www.atgc-montpellier.fr/phyml/). The tree was rooted using a midpoint rooting with FigTree software version 1.4.2.

Genetic distances were determined to analyse genetic variability, and these analyses were conducted using the Tajima-Nei model [18] with 1,000 bootstrap replicates. All of the positions with less than 95% site coverage were eliminated. Therefore, fewer than 5% alignment gaps, missing data, and ambiguous bases were allowed at any position. The variation rate among the sites was modelled with a gamma distribution (shape parameter = 0.5). Evolutionary analyses were conducted using MEGA 6.

#### Intrahepatic total HBV-DNA (it-HBV DNA) and cccDNA quantification in liver biopsies

Intrahepatic DNA was extracted from a range of 5–20 mg of liver biopsies using a DNeasy Blood & Tissue Kit (QIAGEN, Hilden, Germany) according to the manufacturer’s instructions. The amount of extracted DNA was then quantified with a Qubit 2.0 fluorometer (Life Technologies, Invitrogen Division, Darmstadt, Germany). The number of extracted hepatocytes was then calculated on the basis of the total amount of extracted DNA, estimating 7 ng of DNA per cell. The intrahepatic total-HBV DNA of each patient was quantified using a modified COBAS^®^ Ampliprep/COBAS^®^ TaqMan HBV test, v2.0 (Roche Molecular System, Inc., Branchburg, NJ, USA), suspending 10 μl of extracted DNA in 790 μl of virus-free serum. The total intrahepatic level of HBV-DNA, expressed as IU/ml by the instrument, was then converted to copies of it-HBV-DNA/1000 hepatocytes, thus calculating the number of cells effectively used for quantification.

Real-time PCR was used to detect and quantitate cccDNA in the liver tissues of chronic hepatitis B patients with a Light Cycler 2.0 (Roche). In brief, primers and a probe for cccDNA that target a unique gap region located between the two direct repeat regions of cccDNA (DR1 and DR2) were used. The primers sequences were Forward, 5’-ACCTCTCTTTACGCGG-3’, and Reverse, 5’-ACAGCTTGGAGGCTTGAA-3’; the HBV probe sequence was 5’-CTCCCCGTCTGTGCCTTCTCATC-3’, and it was conjugated on its 5′ end to the FAM (6-carboxyfluorescein) reporter dye. Real-time PCR amplification was performed with a LightCycler FastStart DNA Master Hybridization Probe kit (Roche Diagnostics) using a 20-μL reaction mixture containing 5 μL of extracted DNA or plasmid standard dilutions, 9.4 μL of water, 2 μL of Master Mix, 1.6 μL of MgCl_2_ (25 mM), 1 μl of probe (300 pM) and 1 μl of each HBV cccDNA primer (10 μM). The thermal cycling conditions were as follows: 10 min at 95 °C, followed by 45 cycles of 10 s at 95 °C and 30 s at 60 °C. Data were analysed with LightCycler software v3.5 (Roche Diagnostics). Real-time PCR evaluation was performed at least in duplicate for the standard dilutions and samples, and the mean value obtained was used for quantitation. For each run, a standard curve was plotted over a 6-log range, from 10 to 10^6^ copies/reaction, by diluting the plasmid standard (pAM6 [ATCC^®^ 45020D^™^ ATCC, Manassas, VA]). Standard curves were considered acceptable if the correlation coefficients (r^2^) were >0.980.

Finally, the obtained intrahepatic cccDNA levels were expressed as copies of cccDNA/1000 hepatocytes by considering the number of detected cccDNA copies and the number of hepatocytes that were calculated to be in the 5 μl of DNA extract used for the Real-Time PCR.

## Statistical analysis

Statistical analyses of data were performed using SPSS software (v19.0; SPSS Inc., Chicago, IL). Data were expressed as medians (interquartile range [IQR]) for continuous variables and as counts and percentages for discrete variables. Chi-Squared Tests of Independence based on a 2x2 contingency table were used for discrete data, while Mann-Whitney tests were used for continuous data. P<0.05 was considered to be statistically significant. Spearman correlation coefficients were used to investigate correlations between virological parameters.

## Results

Data on the study population is summarized in Table 1. Most subjects were male (353, 98.8%), and the median age was 24 years old (IQR 21–28).

**Table 1:** Study population.

Characteristics	N (%)
**Subjects**	358
**Age in years, median (range)(IQR)**	24.7 (21–54)
**Male sex**	353 (98.6)
**Region of origin**	
West Africa	295 (82)
East Africa	42 (11)
Asia	21 (6)
**HIV-positive**	4 (1.1)
**HCV-positive**	8 (2.2)
**VDRL-positive**	4 (1.1)
**QuantiFERON Gold-positive**	99 (27.6)
**HBV-positive**	209 **(**58.2**)**

Of the 358 studied subjects, 94.1% were from Africa and 5.9% from Asia. Most African subjects (87.5%) originated from West Africa, while only 12.5% were from East Africa.

Notably, 58.1% of the patients (208/358) tested were positive for an HBV marker. For the other screening tests, 27.6% of the subjects were positive for the QuantiFERON Gold Test, 2.2% had anti-HCV antibodies, 1.1% had positive VDRL assays, and 1.1% were HIV-positive.

Among the 208 subjects exposed to HBV infection, 154 (74%) were HBsAg-negative/anti-HBc-positive with undetectable serum HBV-DNA, while 54 (25.5%) were HBsAg-positive/anti-HBc-positive with detectable serum HBV-DNA (Table 2).

**Table 2:** Characteristics of HBsAg-positive patients.

**Male, N (%)**	54 (100)
**African nationality, N (%)**	54 (100)
**Median age, years (IQR)**	25 (21–26)
**HBeAg positive, N (%)**^1^	7 (15.2)
**HBsAg positive, N (%)**	53 (98.1)
**Median ALT**^2^**, (IQR)**	29 (24–48)
**Median AST**^2^**, (IQR)**	25 (21–32)
**Median HBV DNA, logIU/mL (IQR)**	3.0 (2.3–3.6)
**Median HBsAg, IU/mL (IQR)**	10311 (2282–20957)
**Genotype A, N (%)**^3^	7 (16.7)
**Genotype E, N (%)**^3^	34 (81.0)
**Genotype D, N (%)**^3^	1 (2.4)
**Genotype not amplified, N (%)**	12 (28.6)

^1^Available for 46 patients;

^2^ ALT = Alanine transaminase, AST Aspartate transaminase;

^3^available for 42 patients.

HBeAg was not detected in 73.6% of HBsAg-positive patients, and 1 subject was HBsAg-negative despite having detectable serum HBV-DNA. None of the patients had a chronic HDV co-infection.

Phylogenetic analysis performed on 42 HBsAg-positive patients with an available RT/HBsAg sequence revealed that HBV genotype E was predominant (34, 80.9%), followed by HBV genotype A (7, 16.7%) and D (1, 2.4%) (Table 2).

### Clinical, virological and biochemical characteristics of patients with chronic HBV genotype E infection

The large majority of CHB patients had an HBV genotype E infection. Most of them (85.3%) were HBeAg-negative/anti-HBe-positive with a median serum HBV-DNA of 3.2 (IQR: 2.7–3.6) logIU/ml and median ALT of 27 (25–52)U/L (Table 3).

**Table 3:** Characteristics of genotype E infected patients.

**Male, N (%)**	34 (100)
**Median age, years (IQR)**	25 (21–28)
**HBeAg positive, N (%)**^1^	4 (12.1)
**HBsAg positive, N (%)**	34 (100)
**Median ALT, IU/L (IQR)**	27 (25–52)
**Median AST, IU/L (IQR)**	24 (21–32)
**Median HBV DNA, logIU/mL (IQR)**	3.2 (2.7–3.6)
**Median HBsAg, IU/mL (IQR)**	10,899 (5,359–20,272)

^1^Available for 33 patients.

According to EASL Clinical Practice guidelines (CPG) [19], 31/34 patients carrying HBV genotype E had enough information to be classified as follows:

- 25 patients in phase 3 of CHB defined by HBeAg-negativity, anti-HBe-positivity, a median (IQR) serum HBV DNA of 2.8(2.4–3.1) log IU/mL, (<2,000 IU/ml) and normal ALT. In this group, as indicated in EASL CPG we also included patients with median (IQR) serum HBV DNA of 3.6 (3.5–3.7) logIU/ml (>2,000-<20,000 IU/ml) and normal ALT.
- 2 patients in phase 2 of CHB defined by HBeAg-positivity, serum HBV DNA levels >20,000IU/ml (8.5 and 8.6 logIU/ml, respectively) and elevated ALT values.
- 2 patients in phase 4 of CHB defined by HBeAg-negativity, detectable anti-HBe, moderate levels of serum HBV-DNA (3.5 and 3.7 logIU/ml, respectively) and fluctuating or elevated ALT values.
- 1 patient in phase 1 of CHB characterized by HBeAg-positivity, very high levels (> 8 log IU/ml) of serum HBV DNA and ALT persistently within the normal range of traditional cut-off values.

One patient was characterized by an atypical HBV profile. Despite the patient had serum HBV-DNA<2000 IU/ml and HBsAg<1000 IU/ml, he was diagnosed with HBV-related HCC, with ALT values five times higher than normal.

All of the patients were naïve to anti-HBV drugs. The patient affected by HCC started tenofovir for treating HBV infection and sorafenib as chemotherapy. He died four months thereafter.

Analyzing overall population of patients with CHB due to genotype E of HBV, Spearman test l revealed a lack of correlation between serum HBsAg and HBV-DNA levels (rho = 0.17; p = 0–35). Similar result were obtained considering only patients (n = 25) in the phase 3 of CHB (rho = -0.16, p = 0.47) (data not shown).

**Fig 1:**
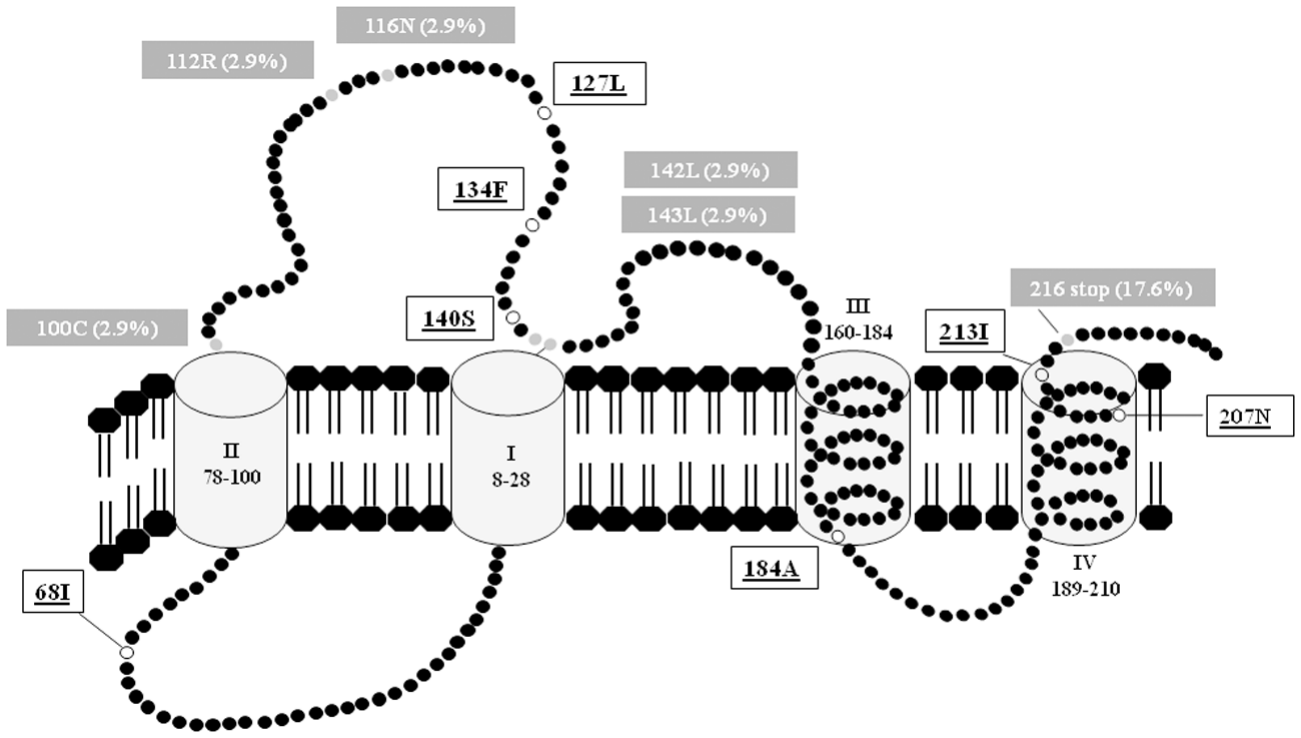
Putative structure of HBsAg reporting the immune-escape mutations and stop-codons (grey box) and HBsAg positions with wild-type amino acids different from genotype D (white box) observed in the group of 34 HBV genotype E infected patients.

The analysis of RT sequences of E genotype viral strains obtained from the 34 CHB patients did not reveal the presence of any primary or compensatory drug-resistance mutations. In 2 patients, mutations at RT position 207 and 215 were identified. In analysing the HBsAg sequences, the presence of a Serine at HBsAg position 140 was detected in all of the patients (Fig 1).

Additional immune escape mutations were detected in 3 patients: T116N, Y100C, and P142L+S143L (Fig 1). Notably, T116N introduces an additional N-linked glycosylation site in HBsAg (Fig 1).

The analysis of the HBsAg sequence in 1 patient with atypical positivity for both HBsAg (31,202 IU/ml) and anti-HBs (101 mIU/ml) revealed the presence of a G112R substitution, which is known to introduce a positively charged amino acid in the first loop of HBsAg, thus potentially hampering binding between HBsAg and anti-HBs (Fig 1).

The presence of stop codons was detected in HBsAg at position 216 in 6 patients (18%) (Fig 1).

### Virological and histological characterization of liver tissue

Nine (26.5%) of the 34 patients with CHB E genotype infection underwent a successful subcostal, ultrasound guided, liver biopsy. Histological staging showed an Ishak score of 0 and 1 in 5 and 4 patients, respectively.

The quantification of total intrahepatic HBV-DNA and cccDNA as well as RT/HBsAg sequences were available only in 5 of these patients; for the other 4 patients the analysis was not possible due to scarcity of the collected liver material (2 cases) or to technical difficulties in extracting DNA (2 cases). All the 5 patients were in the phase 3 of CHB, as they resulted HBe Ag negative, had median (IQR) serum HBV-DNA of 2.4 (2.0–3.0) log IU/ml (<20,000 IU/ml), normal transaminases, no evidence of hepatic necro-inflammatory activity and a minimal degree of fibrosis, as highlighted by liver biopsies.

**Fig 2:**
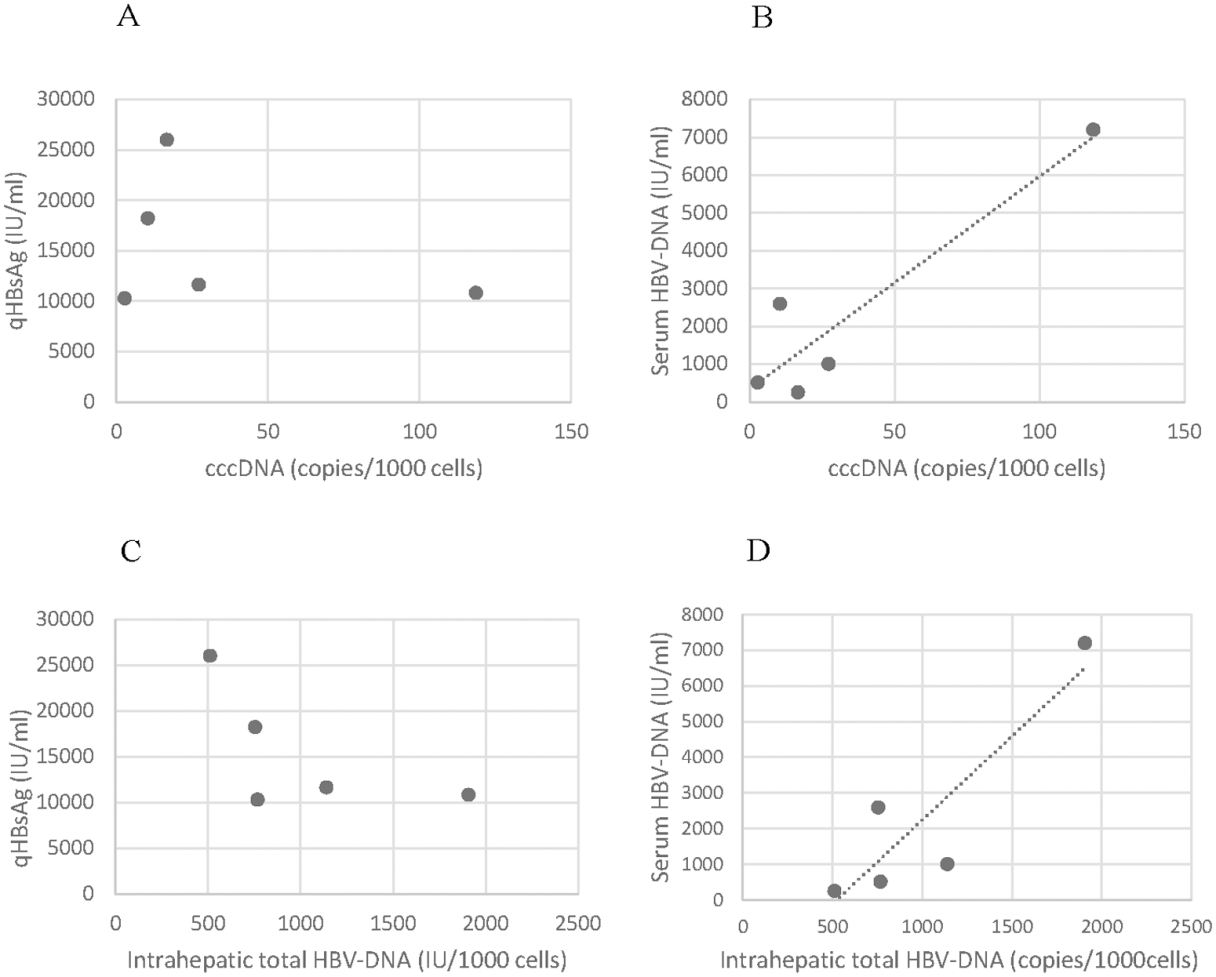
Graphs reporting the correlation between cccDNA and HBsAg titer (A) and serum HBV-DNA (B), and between intrahepatic total HBV-DNA and HBsAg titer (c) and serum HBV-DNA (D) in the 5 HBV genotype E infected patients with available liver biopsies.

The median (IQR) total intrahepatic HBV-DNA was 766 (753–1139) copies/1000 cells, and the median (IQR) cccDNA was 17 (10–27) copies/1000 cells. A trend of correlation was observed for both the total intrahepatic HBV-DNA and cccDNA with serum HBV-DNA (Fig 2).

Conversely, these two parameters did not correlate with the HBsAg titres (Fig 2).

**Fig 3:**
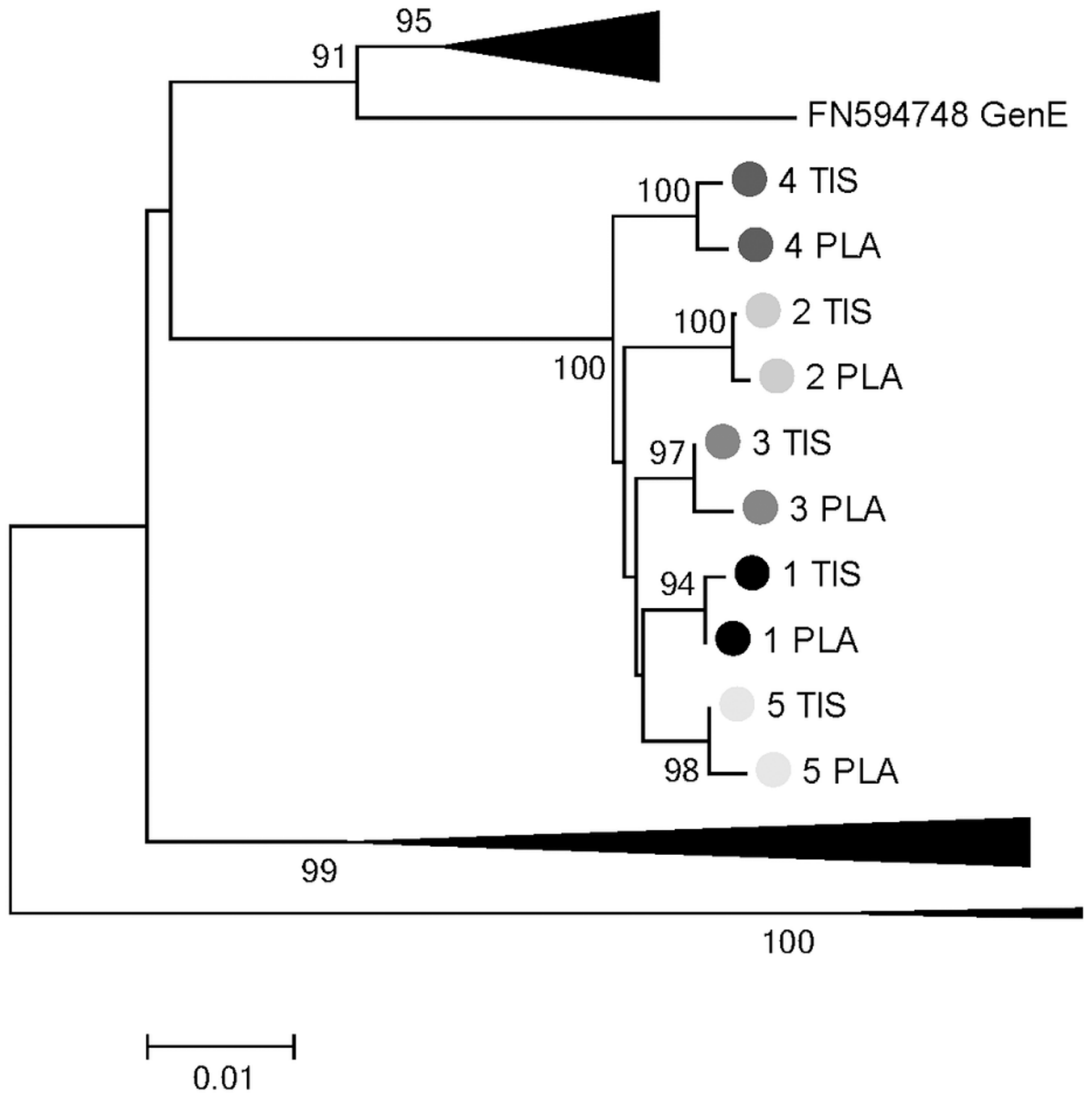
Phylogentic tree reporting the clustering of HBsAg sequences from plasma and liver tissue obtained from the 5 HBV genotype E infected patients with available liver biopsies. Phylogenetic tree was built by using HBsAg sequences from plasma and liver tissue obtained from the 5 HBV genotype E infected patients, and HBsAg sequences from HBV genotype E isolated from Genbank (Accession number:FN594748).

The final step of this study was to compare the extent of genetic variability in the HBsAg sequences obtained in plasma and liver tissue samples. The rate of nucleotide substitutions was 14/1,000 nucleotides in plasma and 12/1,000 nucleotides in liver tissue. By calculating the inter-group genetic distances, we found that HBsAg sequences in plasma and in liver tissue differ by a rate of 2.5/1,000 nucleotides. In particular, in 3 patients, different profiles of nucleotide or amino acid substitutions were identified, suggesting a potential anatomical compartmentalization of viral strains circulating in plasma and those present in liver (Fig 3).

In patient 4 of Fig 3, the HBsAg mutation S204N was detected only in plasma and not in the liver. A similar situation was observed also for patients 2 and 3, and it was characterized by specific mutational profiles (L49R+S193L and G102A/G+S193S/L+S210S/R+L216 stop) observed only in plasma and not in liver tissue (Fig 3). Conversely, in patients 1 and 5 only nucleotide substitutions were identified (Fig 3). A similar situation was observed for the RT sequences.

## Discussion

This study documents a significant rate of HBV infection in a population of immigrants from highly endemic areas of Western and Eastern Africa, 58% of which demonstrated serological markers of a past or ongoing HBV infection. HBV genetic analysis conducted in the CHB subjects revealed that most of the migrants (>80%) were infected with the HBV E genotype, and RT sequencing analysis of the viral strains obtained showed a high prevalence of mutations correlated with immune escape phenomena (Echevarria et al., JMV 2006). Stop codons at HBsAg position 216 were also detected in 18% of the studied patients.

Coppola and colleagues [20] documented an anti-HBc prevalence of >50% among migrants and refugees in southern Italy, reflecting the large spread of HBV infection in their countries of origin. Our results revealed a prevalence (>15%) of CHB higher than that reported by Coppola, who described an HBsAg prevalence ranging from 7% to 14% among African migrants in southern Italy. These results indicate that immigration from countries with high HBV endemicity to those with low HBV endemicity may represent an avenue by which HBV infection can spread. The recent recommendations of the Centers for Disease Control and Prevention, as well as the WHO guidelines for HBV and HCV testing, suggest universal screening for HBV infection in people originating from countries with HBsAg prevalences >2%, as is the case in Africa [21,22]. Universal screening could help intercept CHB migrants who would benefit from antiviral treatments, and it would also be advantageous for HBV-susceptible foreign subjects (roughly 40% in our study) who would benefit from HBV immunization programmes, which strongly reduce the risk of HBV infection and transmission. These considerations are even more relevant when recent publications that have demonstrated the cost-effectiveness of CHB screening, even in low-prevalence populations (e.g., as low as 3%) in the United States, are considered [23–26].

All patients infected with HBV-genotype E were from Western or Eastern Africa, where this genotype is predominant [27–30]. The HBV E genotype is still poorly studied, and it seems to have potential oncogenic activity [31]; however, the mechanisms underlying the oncogenic potential of the HBV genotype E have not yet been clarified. Here, we showed that the majority of CHB E genotype patients had low amounts of serum HBV-DNA and high HBsAg levels, suggesting a viral particle production pathway that is not linked to HBsAg secretion. Unlike to other genotypes [32], we found that HBV genotype E infection was characterized by high serum HBsAg titres that do not correlate with serum HBV-DNA levels (Rho = 0.14, P = 0.44). Recent findings have shown that detectable amounts of HBsAg in serum can derive not only from the transcriptional activity of cccDNA but also from ORF S integrated into the human genome [33]. Although further studies are necessary, it can be hypothesized that HBV genotype E is characterized by a higher rate of ORF S integration, which might explain the higher HBsAg levels (despite low serum HBV-DNA) observed in patients with this infection and could be correlated with an increased risk of HCC onset (in the absence of liver damage). Furthermore, the correlation of HBV genotype E with an increased risk of HCC onset could also be explained by the substantial proportion of patients harbouring a viral strain with a premature stop codon in HBsAg. The presence of stop codons can favour the production of a truncated HBsAg, which accumulates in the endoplasmic reticulum and induces oxidative stress, somehow enhancing cell proliferation. This highlights the need to perform studies on a large cohort to unravel the pro-oncogenic properties of HBV genotype E.

All of the HBV genotype E strains obtained in our study had a Serine at HBsAg position 140, which is known to affect HBsAg recognition by antibodies [34]. Position 140 resides in the HLA class II epitope that encompasses amino acids 139–146 and is known to affect anti-HBs production. This data suggests that HBV genotype E constitutively harbours an amino acid in HBsAg that increases its potential to evade the humoural response and may explain the results from recent studies showing the breakthrough of HBV genotype E infection in vaccinated individuals from West Africa [35]. This data raises the issue of possible HBV genotype E transmission to vaccinated individuals from Western Countries.

In conclusion, a high rate of CHB infection was demonstrated in a group of immigrants from Western Africa in Italy. The large majority of these subjects carried an HBV genotype E strain with serological and virological characteristics of immune escape, which may be the cause of viral replication persistence, the CHB infection process and, ultimately, HCC development. The increasing flow of immigrants from North Africa to Western Countries makes considering the clinical and epidemiological implications of HBV E genotype strain diffusion in low HBV endemic countries critical and highlights the importance of establishing targeted screening programmes and access to care for immigrants to optimize the epidemiological evaluation of these subjects and to establish vaccination programmes as well as to manage chronic HBV genotype E-infected patients, with the aim of reducing their progression to liver cancer.

## Supporting information

S1 File Protocol for population-based sequencing of HBV Reverse Transcriptase/HBsAg. (DOC)
