# Supplementary material for: High rates of chronic HBV genotype E infection in a group of migrants in Italy from West Africa: Virological characteristics associated with poor immune clearance
Source: PLoS One. 2018 Mar 29;13(3):e0195045. doi: 10.1371/journal.pone.0195045 (PMC5875859; doi:10.1371/journal.pone.0195045)
Supplement: S1 File — (DOC) [file pone.0195045.s001.doc]

**S1 file**

**Protocol for population-based sequencing of HBV Reverse Transcriptase/HBsAg.**

HBV-DNA was extracted using a commercially available kit (QIAmp DNA blood mini-kit, Qiagen Inc., USA), and then amplified with Amplitaq-Gold polymerase using the following primer pairs: F1-5’GGTCACCATATTCTTGGGAA and R1-5’GTGGGGGTTGCGTCAGCAAA. Polimerase chain reaction (PCR) conditions were: one cycle at 93 °C for 12 min, 40 cycles (94 °C 50 s, 57 °C 50 s, 72 °C 1 min and 30 s), and a final cycle at 72 °C for 10 min. For samples with serum low HBV-DNA, 2 additional heminested-PCR were performed, starting from the same first amplicon: eminested_1 used the following primer pairs (F1-5’GGTCACCATATTCTTGGGAA and R2-GAGGACAAACGGGCAACATACCTT and eminested-2 used F2-GTTGACAAGAATCCTCACAATA and R1-5’GTGGGGGTTGCGTCAGCAAA. Both eminested-PCRs conditions were: one cycle at 93 °C for 12 min, 35 cycles (94 °C 50 s, 56 °C 50 s, 72 °C 1 min), and a final cycle at 72 °C for 10 min. PCR-products were purified and sequenced by using eight different overlapping sequence-specific primers, a BigDye terminator v. 3.1 cycle sequencing kit (Applied-Biosystems FosterCity, CA) and an automated sequencer (Genetic Analyzer 3130XL). The sequences were analyzed using SeqScape-v.2.5 software. The quality endpoint for each individual gene was ensured by a coverage of the S gene sequence by at least two segments. Sequences having a mixture of wild-type and mutant residues at single positions were considered to have the mutant(s) at that position.
